# Factors affecting faculty conformity in South China universities

**DOI:** 10.3389/fpsyg.2022.923500

**Published:** 2022-08-25

**Authors:** Chuang Xu, Yuan-Cheng Chang

**Affiliations:** ^1^Department of Education Management, Chinese International College, Dhurakij Pundit University, Bangkok, Thailand; ^2^Office of Teaching Quality Supervision and Assessment, Hunan Institute of Technology, Hengyang, Hunan, China

**Keywords:** organizational identification, formalization of organizational structure, conflict management style, faculty conformity, university teachers

## Abstract

Based on social contagion theory, this study examines the mediating role of formalization of organizational structure between organizational identification and faculty conformity. It also analyzes the moderating role of conflict management style between organizational identification and faculty conformity, and formalization of organizational structure and faculty conformity in universities in Hunan province, China. Convenience sampling was employed to select the subjects, and 1,024 Chinese faculty members including teaching staff and administrative staff were surveyed online with the questionnaire consist of organizational identification scale, organizational formalization scale, conflict management style scale, and faculty conformity scale. 1,000 valid respondents were collected and SPSS was used to analyze the data through descriptive analysis, analysis of variance, correlation analysis, and hierarchical multiple regression. The results showed that faculty members’ organizational identification had a positive effect on faculty conformity; formalization of organizational structure partially mediated the relationship between organizational identification and faculty conformity; and conflict management style positively moderated the relationship between organizational identification and faculty conformity and between formalization of organizational structure and faculty conformity. University administrators are often the initiators of conformity as they are responsible for formulating internal regulations. Therefore, they must monitor and coordinate workplace conflicts, resolve and guide faculty conformity, promote individual faculty members’ self-improvement, and foster steady organizational development.

## Introduction

Conformity improves cohesion in an organization, driving members to endorse homogenous values, and work toward shared goals (Burt, 1987; Li and Zhu, 2016). In fact, conformity is a form of social contagion that designates the dissemination of behaviors when individuals come into direct or indirect contact with others (Fenzl and Pelzmann, 2012). In educational institutions like universities, when faculty members display negative behaviors, such as arriving late or leaving early, such attitudes may spread quickly to others. Likewise, when faculty members adopt a positive attitude, such as affability or devotion to work, this behavior may quickly diffuse through the mechanisms of contagion (Jiaqi and Jianfeng, 2019). Padilla-Walker et al. (2013) explains that when the initiator is rewarded or not punished for a certain behavior, the recipient’s imitation is reinforced. This behavioral contagion among faculty members is known as faculty conformity. In educational institutions, when faculty members adopt effective teaching methods, other colleagues often learn from them, especially young faculty members (Berliner, 1986). Faculty members’ careers are increasingly dependent on a culture of progress and achievement (Day, 2002), prompting faculty members who are yet to receive honors to work harder and generate faculty conformity. University faculty members seem less receptive when faced with formal and informal training and learning opportunities, but their participation in training programs is relatively high (Richter et al., 2014). This phenomenon could be explained by the fact that China’s official regulations on teacher training require all teachers to participate in a system of training, fulfilling at least 360 h in a 5-year cycle. Failure to meet the required hours will directly affect their titles and promotion (State Council of China, 2012).

Universities have formal written and explicitly articulated rules and regulations, which are considered as characteristics of formalization of organizational structure. These organizational policies reflect the degree of standardization of work in the organization and the extent to which employee behavior is regulated (Schminke et al., 2002). A formalized organizational structure can constrain faculty members, thereby prompting conformity (Dastmalchian and Blyton, 1998). Examples include the system to track employees’ check-in and check-out when arriving or leaving the workplace and specific methods of classroom management. The theory of inhibitory contagion also implies that the core of conformity is to “ease the feeling of being constrained” (Levy and Nail, 1993). When faculty members are constrained by a formalized organizational structure, they conform to the regulations and comply with the decision of those formulating the regulations, thus reducing the likelihood of “feeling constrained” and generating a contagious mechanism of faculty conformity (Jiaqi and Jianfeng, 2019).

Ferguson (2006) explains that conformity arises due to serious conflict within an individual. Such a conflict can be divided into two types. First, the impulse is strong enough to motivate people to successfully achieve. Second, internal control is strong enough to inhibit such achievements (Jiaqi and Jianfeng, 2019). Individuals have certain tendencies or reactions when dealing with conflicts, known as “conflict management style” (Wee et al., 2021). Positive conflict management style correlates positively with employee discipline through the formalization of organizational structure and organizational behavior (Soieb et al., 2013). Formalization of organizational structure can exacerbate or mitigate conflict and influence individual behavior (Pelled et al., 1999). A compromising conflict management style is more likely to produce conformity (Petersen and Ford, 2019). University faculty members usually adopt the collaborating conflict management style when confronted with conflicts (Williams-Ilemobola et al., 2021), especially when various codes of faculty behavior are included in the formalization of organizational structure, which minimizes conflicts and produces faculty conformity with shared goals (Aditya and Setyawan, 2021). Briefly, conflict management style may have a moderating effect on the formalization of organizational structure and faculty conformity. Furthermore, Bilgicer et al. (2015) highlight that behaviors in the formalization of organizational structure are more contagious than informal behaviors in organizations. Specifically, in conflict management style, as individuals interact constantly with the group, formalization of organizational structure will more likely produce conformity. Levy and Nail (1993) emphasize that conformity is the result of group–individual interactions.

Individual factors are important in predicting conformity (Ferguson, 2006), as it entails diffusion of attitudes or behaviors and leads to social impact and transmission of information or behaviors in this process (Levy and Nail, 1993). Identification is an attitude, or an internal process that maintains relationships with the group or intervenes in an individual’s attitudes (Wu et al., 2022). Thus, the higher the organizational identification, the more likely it will produce conformity (Paolella and Syakhroza, 2021). When faculty members identify with the organization they serve, they incorporate organizational values and cultural goals into their personal objectives, internalize various behavioral codes in the formalization of organizational structure, and produce behaviors of faculty conformity that are consistent with organizational goals (De Cremer and Tyler, 2005; Wu et al., 2022). Maraghoush et al. (2021) argued that organizational identification positively influences normative and consistent ethical behaviors that are constrained by the environment and cognitive perceptions. The formalization of organizational structure has a significant positive effect on normative faculty conformity (Borry et al., 2018). In other words, organizational identification may influence faculty conformity through the formalization of organizational structure. Furthermore, Burt (1987) believes that conformity is determined by interpersonal patterns, and organizational identification represents the interactions and connections in interpersonal relationships between individuals and groups (Wu et al., 2022). It is through interpersonal interactions with others that individuals contribute to the resolution of internal conflicts, which leads to conformity (Jiaqi and Jianfeng, 2019). Mello and Delise (2015) also suggest that conflict management can moderate the relationship between cognitive diversity and cohesion. Similar to organizational identification, cognitive diversity is a concept about attitudes and values (Kilduff et al., 2000), and conformity is a form of cohesion (Carron et al., 2002). Thus, conflict management styles may moderate the relationship between organizational identification and faculty conformity.

The above discussion shows that organizational identification, formalization of organizational structure, and individual conflict management styles of university faculty members determine faculty conformity. However, the influential mechanism between them remains unclear. Clarifying how formalization of organizational structure, individual conflict management styles, organizational identification influence faculty conformity is of great significance to the deepening of conformity theory. Moreover, figuring out (Jiaqi and Jianfeng, 2019) the relationships between the variables and guiding faculty conformity is an important administrative tool to enhance organizational cohesion and accomplish organizational goals (Li and Zhu, 2016), and an effective way to promote individual faculty members’ self-improvement and steady organizational development. Therefore, this study models the relationships among four variables on the basis of the theory of social contagion and uses regression analysis to validate the model to promote and enrich the application of the theory of social contagion in the field of education.

## Literature review and hypothesis development

### Organizational identification and faculty conformity

Organizational identification is a critical factor that binds organizational members and ensures a high level of organizational commitment (Demir, 2015). When individuals identify with an organization, they become cognitively interconnected and develop a sense of belonging with the group (Mael and Ashforth, 1992). This sense of belonging motivates people to integrate group and individual interests, thus triggering the participation of non-direct stakeholders and generating conformity (Klandermans, 2002). Studies also suggest that when a large number of organizational members identify with the organization, their expectations are consistent and they are likely to develop conformity (Paolella and Syakhroza, 2021). Abbasi et al. (2021) also suggest that organizational identification has a significant positive effect on behavior (Demir, 2015; Sharma, 2021), leading to the following hypothesis:

*H1:* Organizational identification has a significant effect on faculty conformity.

### Mediating role of formalization of organizational structure between organizational identification and faculty conformity

Schminke et al. (2002) define the formalization of organizational structure as the extent to which work is standardized in an organization and employee behavior is governed by rules and procedures, with an emphasis on accepted and explicit rules that are documented in the written form. In the setting of schools, it refers to various regulations and rules explicitly articulated in the written form. According to Dastmalchian and Blyton (1998), rules and regulations can be categorized as control rules to regulate and control the behavior of general employees, such as performance appraisal, work attendance, and leave approval; and safeguarding rules for administrators to clarify their responsibilities and prevent them from making arbitrary decisions or taking action that could harm the rights and interests of the organization or employees, such as departmental responsibilities, recruitment procedures, hazard recognition, promotion system, and research management methods. Miles (2012) explains that formalization of organizational structure places constraints on organizations, compelling those established in the same institutional domain and influenced by similar external institutional factors to become homogeneous. This process is the outcome of the impact on individual, organizational, and interorganizational levels (Miles, 2012). That is, within schools, formalization of organizational structure also creates organizational constraints for members, resulting in faculty conformity. Maraghoush et al. (2021) indicate that organizational identification impacts ethical behavior, namely normative and consistent behaviors governed by the environment and cognition. Similar to formalization of organizational structure, organizational identification constrains the behavior of members of an organization. Diminishing the perception of being constrained is a central element in generating faculty conformity (Levy and Nail, 1993). Organizational identification provides individuals with normative guidance and internalizes organizational rules and regulations (Pagliaro et al., 2018), while formalization of the organizational structure affects faculty conformity (Borry et al., 2018; Li et al., 2021). In other words, organizational identification generates faculty conformity through a formalized organizational structure. Therefore, this study proposes the following hypothesis:

*H2:* Formalization of organizational structure has a mediating role between organizational identification and faculty conformity.

### Moderating role of conflict management style

Böhm et al. (2020) propose that conflict entails a relationship between two or more social units, such as individuals, groups, and organizations. Conflicts occur within organizations at four levels: intra-individual, interpersonal, intra-group, and inter-group (Williams-Ilemobola et al., 2021). Conflict management style is an individual’s tendency and reaction when dealing with disputes (Wee et al., 2021). In the developed countries of the West, people are inclined to collaborate and negotiate to resolve conflicts (Shih and Susanto, 2010; Pinto-Moreira, 2021). The avoiding and accommodating styles of conflict resolution predict behavior, but they have a less dominant role (Trudel and Reio, 2011), and the compromising style is more likely to produce conformity (Petersen and Ford, 2019). In China, where collectivism is central to the Asian culture, people are more concerned with their image and relationships, and often adopt avoiding or collaborating styles during conflicts (Hwang, 2000). Adopting a compromising and collaborating style during conflicts can help maintain or protect mutual relationships and produce conformity with shared goals (Williams-Ilemobola et al., 2021).

Social contagion theory indicates that interpersonal patterns are a decisive factor of conformity (Burt, 1987). Organizational identification is an interpersonal pattern in individuals’ interactions with others (Wu et al., 2022). The resolution of conflicts between individuals is facilitated by others (Jiaqi and Jianfeng, 2019). Conformity results from interactions that happen between individuals and groups (Levy and Nail, 1993). In universities, when individuals differ in their opinions or behaviors with their colleagues during performance assessment or teaching reform, if all fellow colleagues believe it is reasonable or unreasonable, individuals will gradually show understanding and agreement with other coworkers. Faculty conformity is generated when faculty members change their own behaviors due to their interactions with others, (Jiaqi and Jianfeng, 2019). Conflict management can moderate the relationship between cognitive diversity and cohesion (Mello and Delise, 2015). Cognitive diversity is a concept about attitudes and values (Kilduff et al., 2000), identification is the representation of attitudes (Wu et al., 2022), and conformity is a form of cohesion (Carron et al., 2002). In other words, conflict management style moderates the relationship between organizational identification and faculty conformity. In addition, Soieb et al. (2013) state that a positive conflict management style is clearly associated with employee discipline in the formalization of organizational structure and organizational behavior. Internal conflicts between recipients can predict conformity (Redl, 1949), where individuals have a strong urge for something but are meanwhile pressured not to act to satisfy that urge in the interim. This may be peer pressure from other members of the organization, and the individual is likely to adopt a collaborating style to satisfy such needs (Jiaqi and Jianfeng, 2019). The pressure may be due to organizational rules and regulations regarding formalization of organizational structure, and the individual adopts an avoidance style that suppresses demands and minimizes conflicts (Aditya and Setyawan, 2021), thereby producing conformity (Petersen and Ford, 2019). Formalization of organizational structure may exacerbate or mitigate conflicts. Conflict management style is a behavioral model for dealing with disagreements. The interaction between the two affected individuals’ behavior and performance (Pelled et al., 1999). Thus, conflict management style may moderate the relationship between the formalization of organizational structure and faculty conformity. As internal behavioral codes in universities are largely developed by administrators, who are also the initiators of contagious behaviors, the contagion exerts a greater impact on general faculty members (Berliner, 1986). Bilgicer et al. (2015) found that behaviors with higher values in organizations are more contagious. Clearly, the formalization of organizational structure involves higher behavioral preferences and legitimacy (Borry et al., 2018). As behaviors on behalf of groups have greater contagion than other behaviors (Ferguson, 2006), this study proposes the following hypotheses:

*H3:* Conflict management style has a moderating effect on organizational identification and faculty conformity.

*H4:* Conflict management style has a moderating effect on formalization of organizational structure and faculty conformity.

## Materials and methods

### Research framework

**Figure 1:**
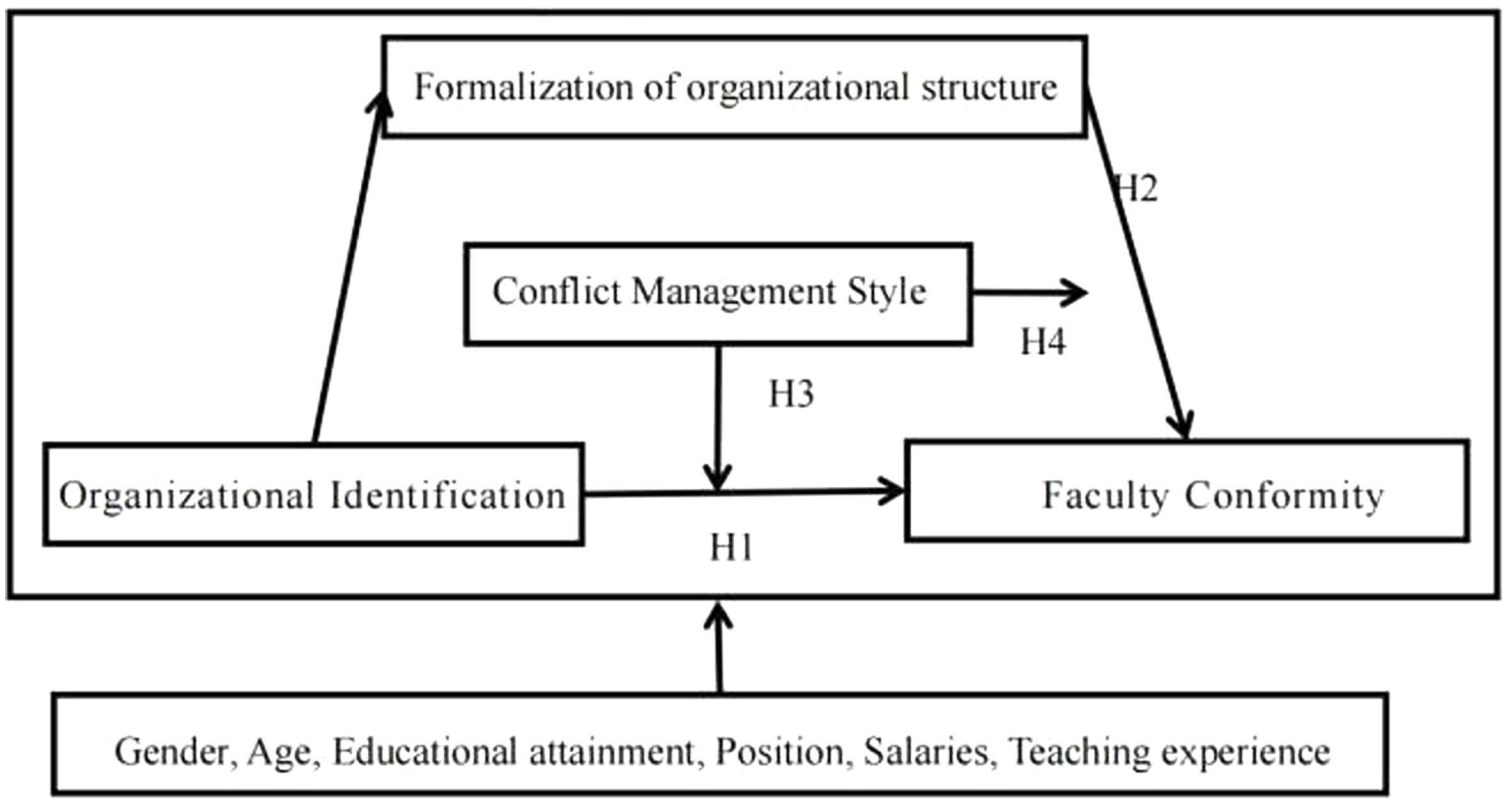
Research framework.

Based on social contagion theory, this study examines whether organizational identification of university faculty members influences faculty conformity through the mediating role of formalization of organizational structure, and whether conflict management style has a moderating effect between organizational identification and faculty conformity, formalization of organizational structure and faculty conformity. A regression analysis was used to validate the study’s theoretical model (Figure 1).

### Research subjects

Convenience sampling was used to select study subjects from four universities that offer undergraduate programs with similar rankings in Hunan Province. The only criterion for inclusion for the sample was being the official faculty members in universities for more than 1 year. Thus teaching staff including professors, associate professors, lecturers and teaching assistants, along with administrative staff including department directors, college deans, and etc. were both included in the study, considering gender, age, educational background, position, salaries and teaching experience as demographic variables. After the pre-survey, questionnaires were distributed by private mails and completed online during the holiday period from October 12 to December 21, 2021. A total of 1,024 questionnaires were distributed, and 24 invalid questionnaires were excluded, with the valid response rate being 97.65%. The study purpose was explained in detail to the participants and signed informed consent was obtained online prior to completing the questionnaire. The collected data were used only for this study and will not be used for other purposes to guarantee participants’ privacy. During the course of this study, we also ensured that participants had the right to withdraw their data at any stage.

### Research tools

Questionnaires offer an objective means of collecting information about people’s knowledge, beliefs, attitudes, and behavior (Boynton and Greenhalgh, 2004). Anonymity ensures the objectivity with the respondents being not disturbed by others when completing the questionnaire. With the development of technology, the online survey with its convenience of access to unique populations, saving time and cost, was widely used in various research fields (Wright, 2005). The online survey also provided the possibility of conducting the research with the limitations of interpersonal communication in the epidemic period regulated by local government (People’s Government of Hengyang, 2021). Besides, the research goal is to uncover the behavioral characteristics of individuals and groups in universities, thus survey method is more suitable.

Organizational identification was measured using the Organizational Identification Scale developed by Mael and Ashforth (1992), a uni-dimensional measurement scale with six questions. For example, “When I hear people praise my school, I feel as if they are praising me.” Likert five point scale from “strongly disagree” to “strongly agree” was adopted, measuring from 1 to 5. The reliability of the original scale was 0.87. After item analysis and exploratory factor analysis, all items are suitable，and the pretest reliability was 0.891. Confirmatory factor analysis (CFA) of the formal survey showed that the factor loadings ranged from 0.79 to 0.86. The construct reliability (CR) was 0.925, higher than the assessment criterion of 0.7; the average variance extracted (AVE) was 0.673, higher than the assessment criterion of 0.5 (Fornell and Larcker, 1981).

The formalization of organizational structure was measured with the Formalization Scale developed by Schminke et al. (2002), a uni-dimensional measurement scale with five questions. For example, “My school has a large number of written rules and regulations.” Likert five point scale from “strongly disagree” to “strongly agree” was adopted, measuring from 1 to 5. The reliability of the original scale was 0.73. After item analysis and exploratory factor analysis, all items are suitable, and the pretest reliability was 0.779. CFA of the formal survey showed that factor loadings ranged from 0.81 to 0.85. The CR was 0.920, higher than the assessment criterion of 0.7; the AVE was 0.696, higher than the assessment criterion of 0.5 (Fornell and Larcker, 1981).

The study subjects were faculty members in China, therefore, to ensure appropriateness of the measurement scale, we drew on studies related to conflict management style in China and the West and adopted the Conflict Management Style Scale developed by Yongmei et al. (2011), a two-dimensional measurement scale with seven questions on the collaborating style, such as “I try to negotiate with my colleagues to be able to reach a compromise, “and eight questions on the compromising style, such as “I choose to give in and not to fight with my colleagues.” Likert five point scale from “strongly disagree” to “strongly agree” was adopted, measuring from 1 to 5. The total reliability of the original scale was 0.86 and the pretest reliability was 0.897. CFA of the formal survey showed that factor loading for the first question on the collaborating style, “I usually give in to my colleagues, “was below 0.7, and therefore it was removed. The remaining 14 questions had factor loadings ranging from 0.80 to 0.87. The collaborating style of CR was 0.938 and the compromising style of CR was 0.935, higher than the assessment criterion of 0.7, and the collaborating style and compromising style of AVE was 0.684 and 0.671, respectively, higher than the assessment criterion of 0.5 (Fornell and Larcker, 1981).

The measurement scale for faculty conformity was adapted from Xu and Tu (2022) conformity scale, a two-dimensional measurement scale with four questions. For example, “When all my colleagues receive a certain academic achievement or honor, I try to get it too.” Likert five point scale from “strongly disagree” to “strongly agree” was adopted, measuring from 1 to 5. The pretest reliability was 0.848. CFA of the formal survey showed that the factor loadings ranged from 0.77 to 0.83. The CR was 0.884, higher than the assessment criterion of 0.7; the AVE was 0.655, higher than the assessment criterion of 0.5 (Fornell and Larcker, 1981).

To make it more applicable and understandable for Chinese faculty members, the two original English scales were translated into Chinese. Dr. Wang, a translation major at Malaya University, and Dr. Gong, an English major at Hunan Normal University were invited to conduct a two-way translation separately on August 11, 2021, and then a pre-test was conducted after a face-to-face discussion on September 6, 2021.

## Results

After the common method variance test for all items, the frequency test is used to show the situation of demographic variables, the analysis of variance (ANOVA) is used to test the difference of demographic variables on each variable. Then correlation analysis was used to test the correlation degree between two variables, and finally the regression analysis is used to test the influential relationship between variables of conflict management style, organizational identification, formalization of organizational structure, and faculty conformity.

### Common method variance test

We used Harman’s single factor test for assessing common method bias and conducted exploratory factor analysis for each variable. The results showed that the variance explained by the first common factor was 34.875%, which is less than the critical criterion of 40% (Harris and Mossholder, 1996). We derived five factors with eigenvalues greater than 1, which distinguished the two-dimensional conflict management style of avoiding and accommodating, along with the other variables of organizational identification, formalization of organizational structure and faculty conformity. The study data were not significantly affected by the common method bias, and the relationships between the variables found from the data were reliable.

### Descriptive statistics and analysis of variance

Descriptive statistics shows that the proportion of female faculty members was 50.4%, similar to the ratio of male to female faculty members in general higher education institutions (50.17: 49.83) in the Hunan Provincial Statistical Yearbook 2020, and the number of female faculty members was increasing every year. Therefore, the sample data reflect the reality. About 53.6% of the participant faculty members were aged 26–45 years, and 6.2% did not obtain a PhD degree; 79.9% were teaching-track faculty members. The monthly salary of 38.9% of the participant faculty members varied between RMB 8,000 yuan and 10,000 yuan; 44.6% participant faculty members had more than 16 years of teaching experience.

The *t-*test showed that there were significant differences in organizational identification (*t* = 1.979, *p* < 0.05) between participant faculty members of different genders, with males having higher organizational identification than females. There were significant differences in the formalization of organizational structure (*t* = 2.669, *p* < 0.01), conflict management style (*t* = 2.630, *p <* 0.01), and faculty conformity (*t* = 2.701, *p* < 0.01) between faculty members with different levels of education. Participants who had a PhD degree scored higher than those who did not have one.

Analysis of variance showed that participant faculty members of different ages, salaries, and years of teaching experience did not qualify the assumption of homogeneity of variance in Levene’s test (*p* < 0.001) for each variable. ANOVA (*p* < 0.001) showed significant differences between different groups. *Post hoc* tests using Dunnett’s T3 method revealed that participant faculty members aged over 55 years scored significantly higher than faculty members of other age groups on all four variables. Those with salaries of RMB 10,000 yuan or more scored significantly higher on all four variables than those paid less than 4,000 yuan. Participant faculty members with more than 16 years of teaching experiences scored significantly higher on all four variables than those with less teaching experience.

### Correlation analysis

Correlation coefficients ranging from 0.324 to 0.481. The variables moderately correlated one with another, and the correlations were positively significant (*p* < 0.001). The mean values ranged from 3.790 to 3.993, indicating a moderate to high status. Table 1 shows the Cronbach’s alpha of the formal survey.

**Table 1:** Summary of correlation analysis.

Variables	*M* ± SD	Organizational identification	Formalization of organizational structure	Conflict management style	Faculty conformity	Cronbach’s alpha
Organizational identification	3.993 (0.906)	1				0.925
Formalization of organizational structure	3.811 (0.987)	0.324^***^	1			0.919
Conflict management style	3.807 (0.684)	0.480^***^	0.460^***^	1		0.897
Faculty conformity	3.790 (0.914)	0.373^***^	0.378^***^	0.481^***^	1	0.883

*** *p* < 0.001.

### Regression analysis

The hypotheses were tested by regression analysis. Consistent with Cohen et al. (2014), we normalized organizational identification, the formalization of organizational structure, conflict management style, and the normalized scores were multiplied together to evaluate the interaction effect. In addition, we drew on the test for the mediation of a moderator effect proposed by Muller et al. (2005) and Edwards and Lambert (2007).


(M4 in Table 2) (1) $$Y=\beta_{10}+\beta_{11}X+\beta_{12}Mo+\beta_{13}XMo+\varepsilon_{1}$$

(M2 in Table 2) (2) $$Me=\beta_{20}+\beta_{21}X+\beta_{22}Mo+\beta_{23}XMo+\varepsilon_{2}$$

(M5 in Table 2) (3) $$\begin{array}{c} Y=\beta_{30}+\beta_{31}X+\beta_{32}Mo+\beta_{33}XMo\\ +\beta_{34}Me+\beta_{35}MoMe+\varepsilon_{3} \end{array}$$

According to Edwards and Lambert (2007), for this model, the regression equation for M is Equation 4,


(M1 in Table 2) (4) $$Me=\beta_{40}+\beta_{41}X+\varepsilon_{4}$$

Subscripts on regression coefficients indicate the equation in which the coefficient is estimated and the number to which the coefficient is assigned.

If *β*_13_ in Equation 1 is significant, then the moderation occurs in the direct effect path model (Edwards and Lambert, 2007), For Equations 2, 3, if *β*_21_ ≠ 0 and *β*_35_ ≠ 0 or *β*_23_ ≠ 0 and *β*_34_ ≠ 0 or *β*_23_ ≠ 0 and *β*_35_ ≠ 0, then moderated mediation model is established. The results are shown in Table 2.

**Table 2:** Summary of regression analysis of moderated mediation.

	Formalization of organizational structure		Faculty conformity			
	M1	M2	M3	M4	M5	M5 95%CI
Gender	−0.009	−0.000	−0.006	0.004	−0.001	[−0.098, 0.093]
Age	0.120^***^	0.082^**^	0.145^***^	0.1103^**^	0.076^*^	[0.013, 0.085]
Educational attainment	0.0.49	0.038	0.041	0.029	0.028	[−0.046, 0.148]
Position	−0.000	0.013	−0.012	0.002	−0.000	[−0.111, 0.111]
Salaries	0.050^*^	0.050	0.064^*^	0.063^*^	0.052^*^	[0.000, 0.072]
Teaching experience	−0.317^***^	−0.178^***^	−0.249^***^	−0.101^**^	−0.044	[−0.101, 0.025]
Organizational identification	0.194^***^	0.191^***^	0.255^***^	0.244^***^	0.190^***^	[0.105, 0.262]
Conflict management style		0.279^***^		0.305^***^	0.295^***^	[0.190, 0.356]
Organizational identification x conflict management		0.154^***^		0.156^***^	0.105^**^	[0.024, 0.159]
Formalization of organizational structure					0.177^***^	[0.094, 0.237]
Formalization of organizational structure × Conflict management					0.141^***^	[0.062, 0.202]
R2	0.247	0.301	0.252	0.315	0.337	
△ R2	–	0.054	–	0.063	0.022	
F	46.49^***^	47.37^***^	47.79^***^	50.53^***^	45.62^***^	

Numbers are normalized regression coefficients.

* *p* < 0.05;

** *p* < 0.01;

*** *p* < 0.001.

After dummy coding the demographic variables, age, salary, and teaching experience were significant in predicting faculty conformity, with age having a significant positive effect on the formalization of organizational structure and teaching experience showing a negative relationship with the formalization of organizational structure, consistent with Maurizio (2014) study.

Organizational identification significantly predicted faculty conformity (*β* = 0.255, *p* < 0.001, M3 in Table 2), consistent with Paolella and Syakhroza (2021); thus, H1 is supported. Regression analysis of moderated mediation shows that the interaction between organizational identification and conflict management style shows faculty conformity (*β* = 0.105, *p* < 0.01, M5 in Table 2); thus, H2 and H3 is supported. As Norman et al. (2005) argue, the interaction between organizational identification and conflict management style is a sufficient condition for triggering contagion. The interaction between formalization of organizational structure and conflict management style indicates faculty conformity (*β* = 0.141, *p* < 0.001, M5 in Table 2); thus, H4 is supported.

In addition, the mediation effect of formalization of organizational structure between organizational identification and faculties’ conformity behavior was tested by Sobel test, *z* = 4.816 (*P* < 0.001), which means the mediation effect was significant (Sobel, 1982).

**Figure 2:**
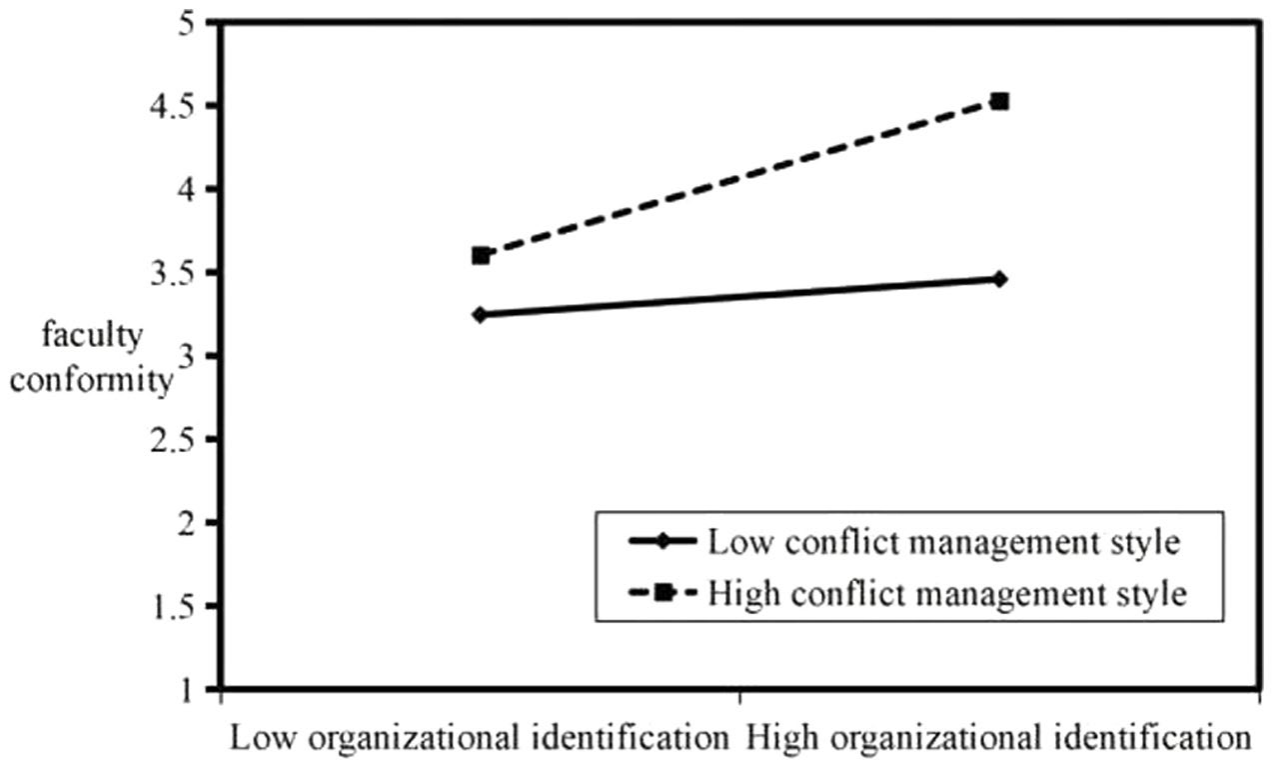
Moderating effect of conflict management style on the relationship between organizational identification and faculty conformity.

**Figure 3:**
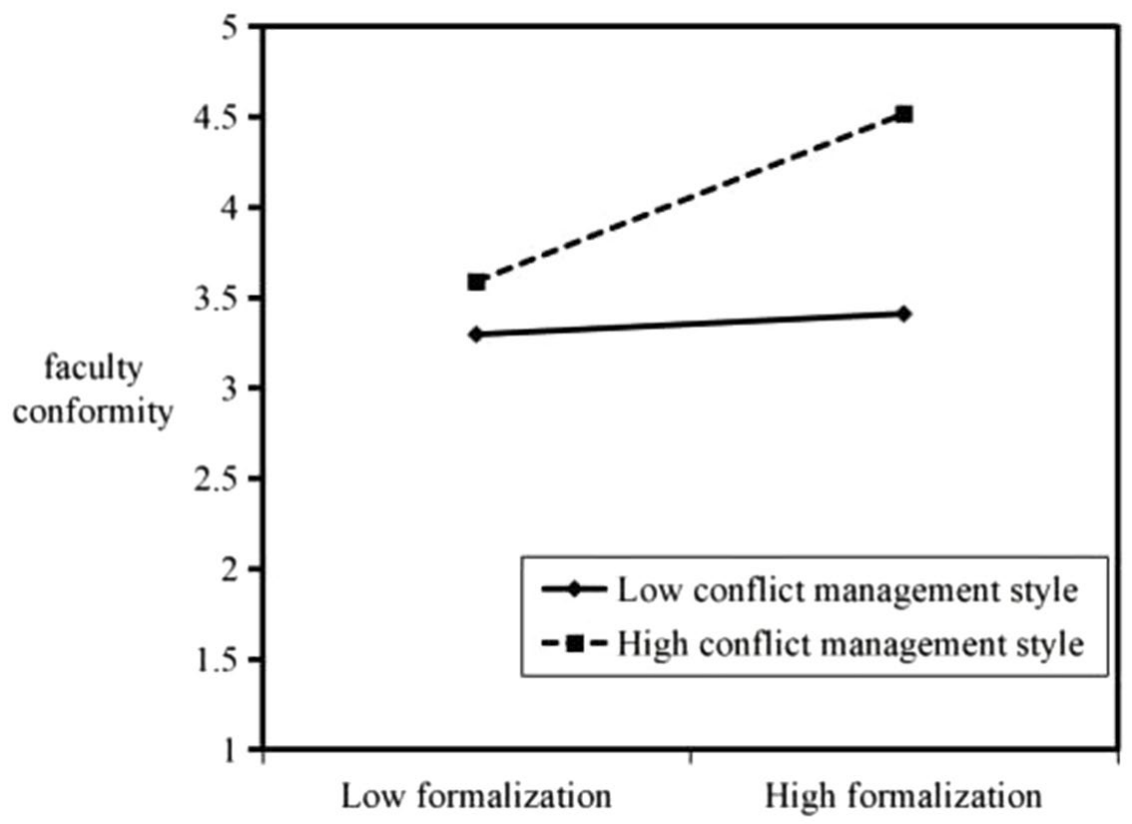
Moderating effect of conflict management style on the relationship between formalization and faculty conformity.

The direction of the interaction effect is clearly plotted and shown in Figures 2, 3. When individual faculty members have a positive conflict management style, organizational identification and formalization of organizational structure are effective in enhancing faculty members’ willingness to engage in conformity. Similarly, when individual faculty members have a negative conflict management style, organizational identification, and formalization of organizational structure can enhance faculty conformity; only the frequency will be reduced compared with the case of a positive conflict management style.

## Discussion

Variance analysis shows that faculty members with a PhD degree, more than 55 years old, more than 16 years of teaching experiences, salaries of 10,000 RMB or more, performed better on the four variables compared with faculty members in other groups. Interestingly, in universities, a high level of education indicates better salaries, while those who are older and have more teaching experience indicates higher working age. Working age is directly linked to salaries, and people with higher salaries will show better organizational identification and willingness to stay and serve the organization (Sugirtha et al., 2020). As the years of service increase, the degree of immersion in the organizational culture is higher, which in turn increases organizational identification. In addition, they are more willing to obey the rules and regulations of the organization and complete various tasks, resulting in faculty conformity.

The regression analysis of demographic variables shows that age has positive organizational identification and faculty conformity. In this study, 53.6% of young faculty members completed their transition from being a student to becoming a teacher, but their place of study or work did not change—they just moved from one university to another. They were accustomed to and internalized the behavioral constraints associated with the formalization of organizational structure as their own behavioral codes, and their pursuit of progress and achievements also contributed toward faculty conformity (Day, 2002). The number of teaching years negatively affected the formalization of organizational structure (Maurizio, 2014). In this study, 44.6% faculty members had more than 16 years of teaching experience and were less satisfied with the organization than those with less teaching experience, which is attributable to a lack of positive perception of formalization of organizational structure among faculty members with extensive teaching experience (Ma and MacMillan, 1999). As teaching experience increases, faculty members’ tolerance for assessment stipulated in the formalization of organizational structure decreases (Shi et al., 2021), thereby reducing the frequency of conformity (Day, 2002). Therefore, reducing the negative effect of the increase in teaching years is also an important issue that administrators must focus on.

The partial mediating effects showed that organizational identification has a positive impact on formalization of organizational structure (Maraghoush et al., 2021). A higher level of organizational identification is more likely to produce faculty conformity (Paolella and Syakhroza, 2021), and formalization of organizational structure can also contribute to faculty conformity (Borry et al., 2018; Li et al., 2021). This validates the social contagion theory. When faculty members are subjected to behavioral constraints resulting from the process of formalization of organizational structure, they will conform to institutional requirements and comply with the wishes of those formulating the regulations, producing faculty conformity (Levy and Nail, 1993). Improving faculty members’ organizational identification is an effective way to increase faculty conformity. Extensively using the process of formalization of organizational structure can increase the frequency of faculty conformity; however, its specific effect must be considered, such as the phenomenon of high participation but low acceptance of teacher training (Richter et al., 2014).

The moderating effect shows that organizational identification and formalization of organizational structure positively enhance faculty conformity, regardless of the positive or negative conflict management style. Notably, the style of conflict management has a positive effect on faculty conformity (Petersen and Ford, 2019). Positive conflict management reduces employee turnover (De Dreu and Beersma, 2005), indicating that administrators who help faculty members deal promptly with conflicts are more effective in retaining talent. The interaction between organizational identification and conflict management shows that rational use of individual teachers’ strong identification with the organization can develop a positive conflict management style and mitigate intra-individual, interpersonal, intra-group, and intergroup conflicts (Williams-Ilemobola et al., 2021), and thereby generate faculty conformity (Redl, 1949; Norman et al., 2005). It is an effective way for administrators to stimulate faculty members’ compliance with the administration (Pounder, 2003). The interaction between formalization of organizational structure and conflict management shows that bureaucratic solutions can regulate teachers’ behaviors by clarifying responsibilities applying various rules and regulations in the formalization of organizational structure, thereby restraining conflict within the recipients (Wheeler, 1966; Pelled et al., 1999). It is an effective way for administrators to enhance faculty conformity. In addition, Petersen and Ford (2019) emphasize that teacher training is related to personal values and conflict management styles. As opposed to “forced” participation due to institutional requirements in teacher training, administrators can enhance the effectiveness of training by increasing faculty members’ organizational identification, and this is attributable to the fact that faculty members who identify with the organization are more likely to adopt consistent organizational values (De Cremer and Tyler, 2005).

## Conclusion and implication

It is important for university leaders to guide faculty conformity behavior in order to condense the organizational centripetal force and achieve organizational goals (Li and Zhu, 2016). It is also an effective way to promote the individual improvement of faculty members and the steady development of the organization. This study shows that faculty members with higher with the organizational identification will have a higher frequency of conformity behavior. The ways to improve faculty members organizational identification can be started from encouraging them to improve their education, increasing their salaries, recruiting more excellent young teachers, and so on. The formalization of organizational structure can also restrict faculty members’ behavior and produce conformity behavior that meets organizational goals. However, with the increase of working years, the constraints of formal rules will be weaken. Organizational identity can obviously alleviate this phenomenon.

In addition to the influence of organizational identification and formalization of organizational structure on faculty members’ conformity behavior, the role of conflict management style in the variable model of this study has also been confirmed. Positive conflict management style is obviously more important when solving the intra-individual, interpersonal, intra-group, and inter-group conflict problems. Therefore, university leaders should adopt some intervention strategies on faculty members with low frequency by improving their organizational identification and promoting positive conflict management style. The positive conflict management style is more conducive to easing the sense of restraint brought by the rules and regulations in the organization, which are indispensable and necessary for administration.

In universities, education, titles, and positions are directly related to faculty members’ salaries, and a higher level of education is relevant to the evaluation of titles. Distinct from their titles and positions, individuals have full control over the level of education that they can strive to obtain. Given the findings of this study, administrators can encourage faculty members to improve their educational attainment and raise their salaries for the purpose of retaining talent (Sugirtha et al., 2020). In addition, young faculty members appear to be more willing to comply with regulations in the formalization of organizational structure (Ma and MacMillan, 1999), which produces faculty conformity. Administrators responsible for human resources management can introduce more young faculty members in universities, consistent with the proportion of young faculty members, to stimulate organizational dynamics.

The success and stability of an organization depends on the ability of its managers to identify and manage workplace conflicts (Doherty and Guyler, 2008). Administrators, who are responsible for the formulation of internal regulations, should make full use of the normative nature of regulations to effectively “discipline” faculty behavior, especially at the beginning of implementing regulations (Trudel and Reio, 2011), and adopt a collaborative conflict management style to minimize internal conflicts and generate positive faculty conformity (Aditya and Setyawan, 2021). Faculty conformity must be monitored as a part of their daily routine and make complete use of the interaction between individuals, groups, and departments to effectively resolve conflicts and stimulate conformity (Williams-Ilemobola et al., 2021). In addition, administrators are also the initiators of contagious behaviors; therefore, it is important for them to promote positive energy through mechanisms of social contagion (Jiaqi and Jianfeng, 2019). This could promote self-improvement among individual faculty members and steady organizational development.

There are some limitations in this study. It is difficult to collect data when subordinates are expected to complete questionnaires about their superiors in universities that are governed or administered in a bureaucratic style. We encountered this problem during the pre-survey. This may be because subordinates are reluctant to challenge their superiors in any manner due to the fear of negative consequences such as losing their jobs (Holt and DeVore, 2005). Therefore, this study has only incorporated the assessment of formalization of organizational structure without extending the research to conformity between their superiors and subordinates (Zhang et al., 2018). We suggest that future studies may include a cohort analysis of administrators and non-administrators. In addition, the faculty conformity scale used in this study does not distinguish between faculty members’ behaviors in teaching and administrative work and does not include the case of negative behaviors of conformity. Therefore, future studies may increase the dimensions of this scale or adopt a more mature scale.

## Data availability statement

The raw data supporting the conclusions of this article will be made available by the authors, without undue reservation.

## Author contributions

CX conducted the study and drafted the manuscript. Y-CC participated in the design of the study and helped to revise the manuscript. All authors contributed to the article and approved the submitted version.

## Conflict of interest

The authors declare that the research was conducted in the absence of any commercial or financial relationships that could be construed as a potential conflict of interest.

## Publisher’s note

All claims expressed in this article are solely those of the authors and do not necessarily represent those of their affiliated organizations, or those of the publisher, the editors and the reviewers. Any product that may be evaluated in this article, or claim that may be made by its manufacturer, is not guaranteed or endorsed by the publisher.

## References

[ref1] Abbasi S. G. Shabbir M. S. Abbas M. Tahir M. S. (2021). HPWS and knowledge sharing behavior: The role of psychological empowerment and organizational identification in public sector banks. J. Public Aff. 21:e2512. doi: 10.1002/pa.2512

[ref2] Aditya S. Setyawan A. (2021). Conflict management and job satisfaction in Indonesia's public organization. J. Int. Bus. Manag. 4, 1–14. doi: 10.37227/JIBM-2021-01-134

[ref3] Berliner D. C. (1986). In pursuit of the expert pedagogue. Educ. Res. 15, 5–13. doi: 10.3102/0013189X015007007

[ref4] Bilgicer T. Jedidi K. Lehmann D. R. Neslin S. A. (2015). Social contagion and customer adoption of new sales channels. J. Retail. 91, 254–271. doi: 10.1016/j.jretai.2014.12.006

[ref5] Böhm R. Rusch H. Baron J. (2020). The psychology of intergroup conflict: a review of theories and measures. J. Econ. Behav. Organ. 178, 947–962. doi: 10.1016/j.jebo.2018.01.020

[ref6] Borry E. L. DeHart-Davis L. Kaufmann W. Merritt C. C. Mohr Z. Tummers L. (2018). Formalization and consistency heighten organizational rule following: experimental and survey evidence. Public Adm. 96, 368–385. doi: 10.1111/padm.12407

[ref7] Boynton P. M. Greenhalgh T. (2004). Selecting, designing, and developing your questionnaire. BMJ 328, 1312–1315. doi: 10.1136/bmj.328.7451.1312, PMID: 15166072 PMC420179

[ref8] Burt R. S. (1987). Social contagion and innovation: cohesion versus structural equivalence. Am. J. Sociol. 92, 1287–1335. doi: 10.1086/228667

[ref9] Carron A. V. Bray S. R. Eys M. A. (2002). Team cohesion and team success in sport. J. Sports Sci. 20, 119–126. doi: 10.1080/026404102317200828, PMID: 11811568

[ref10] Cohen P. West S.G. Aiken L.S. (2014). Applied Multiple Regression/Correlation Analysis for the Behavioral Sciences. London: Psychology Press.

[ref11] Dastmalchian A. Blyton P. (1998). Organizational flexibility in cross-National Perspective: An introduction. Int. J. Hum. Resour. Manag. 9, 437–444. doi: 10.1080/095851998340991

[ref12] Day C. (2002). The government of the United States of America has been working on a number of initiatives to improve the quality of education in the country. Int. J. Educ. Res. 37, 677–692. doi: 10.1007/1-4020-4773-8_41

[ref13] De Dreu C. K. W. Beersma B. (2005). Conflict in organizations: beyond effectiveness and performance. Eur. J. Work Organ. Psychol. 14, 105–117. doi: 10.1080/13594320444000227

[ref14] De Cremer D. Tyler T. R. (2005). Managing group behavior: the interplay between procedural justice, sense of self, and cooperation. Adv. Exp. Soc. Psychol. 37, 151–218. doi: 10.1016/S0065-2601(05)37003-1

[ref15] Demir K. (2015). The effect of organizational justice and perceived organizational support on organizational citizenship behaviors: the mediating role of organizational identification. Eurasian J. Educ. Res. 15, 131–148. doi: 10.14689/ejer.2015.60.8

[ref16] Doherty N. Guyler M. (2008). The Essential Guide to Workplace Mediation & Conflict Resolution: Rebuilding Working Relationships. London: Kogan Page Publishers.

[ref01] Edwards J. R. Lambert L. S. (2007). Methods for integrating moderation and mediation: a general analytical framework using moderated path analysis. Psychol. Methods 12, 1–22. doi: 10.1037/1082-989X.12.1.1 17402809

[ref17] Fenzl T. Pelzmann L. (2012). Psychological and social forces behind aggregate financial market behavior. J. Behav. Financ. 13, 56–65. doi: 10.1080/15427560.2012.655383

[ref18] Ferguson M. J. (2006). “From bad to worse: a social contagion model of organizational misbehavior,” in *IACM 2006 Meetings Paper*. Philadelphia, USA.

[ref20] Fornell C. Larcker D. F. (1981). Evaluating structural equation models with unobservable variables and measurement error. J. Mark. Res. 18, 39–50. doi: 10.2307/3151312

[ref21] Harris S. G. Mossholder K. W. (1996). The affective implications of perceived congruence with culture dimensions during organizational transformation. J. Manag. 22, 527–547. doi: 10.1177/014920639602200401

[ref22] Holt J. L. DeVore C. J. (2005). Culture, gender, organizational role, and styles of conflict resolution: a meta-analysis. Int. J. Intercult. Relat. 29, 165–196. doi: 10.1016/j.ijintrel.2005.06.002

[ref23] Hwang K.-K. (2000). Chinese relationalism: Theoretical construction and methodological considerations. J. Theory Soc. Behav. 30, 155–178. doi: 10.1111/1468-5914.00124

[ref24] Jiaqi Y. Jianfeng J. (2019). “Theory of social contagion,” in Management and Organization Research. eds. Li C. Xu S. (Beijing: Peking University Press), 372–378.

[ref25] Kilduff M. Angelmar R. Mehra A. (2000). Top management-team diversity and firm performance: examining the role of cognitions. Organ. Sci. 11, 21–34. doi: 10.1177/1059601113493925

[ref26] Klandermans B. (2002). How group identification helps to overcome the dilemma of collective action. Am. Behav. Sci. 45, 887–900. doi: 10.1177/0002764202045005009

[ref27] Levy D. A. Nail P. R. (1993). Contagion: A theoretical and empirical review and reconceptualization. Genet. Soc. Gen. Psychol. Monogr. 119, 233–284. 8405969

[ref28] Li Z. Bao X. Sheng Y. Xia Y. (2021). Research on unsafe behavior of construction workers under the bidirectional effect of formal rule awareness and conformity mentality. Front. Psychol. 12:794394. doi: 10.3389/fpsyg.2021.794394, PMID: 34975693 PMC8717065

[ref29] Li H. Zhu J. (2016). “Destructive leadership, Employees' voice, and organization,” in Diversity of Administratorial Perspectives from Inside China. ed. Mandal P. (Singapore: Springer), 205–221.

[ref30] Ma X. MacMillan R. B. (1999). The government of the United States of America is committed to the development of the education system and to the development of the education system. J. Educ. Res. 93, 39–47. doi: 10.1080/00220679909597627

[ref31] Mael F. Ashforth B. E. (1992). Alumni and their Alma mater: a partial test of the reformulated model of organizational identification. J. Organ. Behav. 13, 103–123. doi: 10.1002/job.4030130202

[ref32] Maraghoush J. Gholamreza J. Y. Atalou H. Avarsin S. M. (2021). The model of the impact of organizational socialization on responsibility and ethical behavior through the mediation of organizational identification: a case study of faculty members. Organ. Cult. Manag. 19, 226–250. doi: 10.22059/jomc.2020.301461.1008027

[ref33] Maurizio R. (2014). Labour formalization and declining inequality in Argentina and Brazil in the 2000s: a dynamic approach. ILO Res. Pap. 9, 1–25. doi: 10.2139/ssrn.2530022

[ref34] Mello A. L. Delise L. A. (2015). Cognitive diversity to team outcomes: the roles of cohesion and conflict management. Small Group Res. 46, 204–226. doi: 10.1177/1046496415570916

[ref35] Miles J. A. (2012). Management and Organization Theory: A Jossey-Bass Reader. Vol.9. Hoboken: John Wiley & Sons.

[ref36] Muller D. Judd C. M. Yzerbyt V. Y. (2005). When moderation is mediated and mediation is moderated. J. Pers. Soc. Psychol. 89, 852–863. doi: 10.1037/0022-3514.89.6.852 16393020

[ref37] Norman S. Luthans B. Luthans K. (2005). The proposed contagion effect of hopeful leaders on the resiliency of employees and organizations. J. Leadersh. Organ. Stud. 12, 55–64. doi: 10.1177/107179190501200205

[ref38] Padilla-Walker L. M. Coyne S. M. Fraser A. M. Stockdale L. A. (2013). Is Disney the nicest place on earth? A content analysis of prosocial behavior in animated Disney films. J. Commun. 63, 393–412. doi: 10.1111/jcom.12022

[ref39] Pagliaro S. Presti A. L. Barattucci M. Giannella V. A. Barreto M. (2018). On the effects of ethical climate (s) on employees' behavior: a social identity approach. Front. Psychol. 9:960. doi: 10.3389/fpsyg.2018.00960, PMID: 29951022 PMC6008529

[ref40] Paolella L. Syakhroza M. A. (2021). Beyond the insider-outsider divide: heterogeneous effects of organizational identity and category taken-for-Grantedness on conformity. Soc. Forces 99, 1487–1517. doi: 10.1093/sf/soaa081

[ref41] Pelled L. H. Eisenhardt K. M. Xin K. R. (1999). Exploring the black box: an analysis of work group diversity, conflict and performance. Adm. Sci. Q. 44, 1–28. doi: 10.2307/2667029

[ref42] People’s Government of Hengyang (2021). “Urgent notice to strengthen control of key public places. Available at: https://www.hengyang.gov.cn/xxgk/bmxxgkml/szfjg/sgaj/tzgg/20210805/i2448453.html. (Accessed October 12, 2021).

[ref43] Petersen B. K. Ford D. P. (2019). Are business students prepared for the world of business? Self-interest, conformity and conflict styles. Can. J. Adm. Sci/Revue Canadienne des Sciences de l'Administration 36, 498–513. doi: 10.1002/cjas.1523

[ref44] Pinto-Moreira P. (2021). Conflict management styles with peers at work: gender and levels of education differences. J. EU Res. Bus. 2021, 1–11. doi: 10.5171/2021.427135

[ref45] Pounder J. S. (2003). Employing transformational leadership to enhance the quality of management development instruction. J. Manag. Dev. 22, 6–13. doi: 10.1108/02621710310454824

[ref46] Preacher K. J. Rucker D. D. Hayes A. F. (2007). Addressing moderated mediation hypotheses: theory, methods, and prescriptions. Multivar. Behav. Res. 42, 185–227. doi: 10.1080/00273170701341316, PMID: 26821081

[ref47] Redl F. (1949). “The phenomenon of contagion and “shock effect” in group therapy,” in Searchlights on Delinquency: New Psychoanalytic Studies. ed. Eissler K. R. (Madison, CT: International Universities Press), 315–328.

[ref48] Richter D. Kunter M. Klusmann U. Lüdtke O. Baumert J. (2014). “Professional development across the teaching career: faculty members' uptake of formal and informal learning opportunities,” in Faculty Members' Professional Development. eds. Krolak-Schwerdt S. Glock S. Böhmer M. (Rotterdam: Sense Publishers), 97–121.

[ref49] Schminke M. Cropanzano R. Rupp D. E. (2002). Organization structure and fairness perceptions: the moderating effects of organizational level. Organ. Behav. Hum. Decis. Process. 89, 881–905. doi: 10.1016/S0749-5978(02)00034-1

[ref50] Sharma A. (2021). Want engaged employees? Encourage human resource and enhance organizational connectedness. Perception 6, 1–12. doi: 10.52283/nswrca.ajbmr.hxnp5021

[ref51] Shi S. Zhang Z. Wang Y. Yue H. Wang Z. Qian S. (2021). The relationship between university faculty members' frustration tolerance and academic performance. Front. Psychol. 12:564484. doi: 10.3389/fpsyg.2021.564484, PMID: 33833704 PMC8021856

[ref52] Shih H.-A. Susanto E. (2010). Conflict management styles, emotional intelligence, and job performance in public organizations. Int. J. Confl. Manag. 21, 147–168. doi: 10.1108/10444061011037387

[ref02] Sobel M. E. (1982). Asymptotic confidence intervals for indirect effects in structural equation models. Sociol. Methodol. 13, 290–312. doi: 10.2307/270723

[ref53] Soieb A. Z. Mohd J. O. D'Silva J. L. (2013). The effects of perceived leadership styles and organizational citizenship behaviour on employee engagement: the mediating role of conflict management. Int. J. Bus. Manag. 8:91. doi: 10.5539/ijbm.v8n8p91

[ref54] State Council of China (2012). Opinions of the state council on strengthening the team construction of faculty members. Available at: http://www.gov.cn/gongbao/content/2012/content_2226134.htm (Accessed December 26, 2021).

[ref55] Sugirtha C. M. R. Hameed S. S. Arumugam T. (2020). The impact of organizational identification and employee engagement on intellectual capital assets: an empirical study. Test Eng. Manag. 83, 6277–6285.

[ref56] Trudel J. Reio Jr T. G. (2011). Managing workplace incivility: the role of conflict management styles-antecedent or antidote? Hum. Resour. Dev. Q. 22, 395–423. doi: 10.1002/hrdq.20081

[ref57] Wee S. X. R. Choo W. Y. Cheng C.-Y. (2021). The influence of tertiary education disciplines on self-construals and conflict management tendencies. Front. Psychol. 12:659301. doi: 10.3389/fpsyg.2021.659301, PMID: 34149548 PMC8212056

[ref58] Wheeler L. (1966). Toward a theory of behavioral contagion. Psychol. Rev. 73, 179–192. doi: 10.1037/h0023023

[ref59] Williams-Ilemobola O. B. Adetayo A. J. Asiru M. A. Ajayi J. L. (2021). Librarians' emotional intelligence and conflict management in private university libraries in south-West and south-south, Nigeria. Informat. Impact 12, 33–46. doi: 10.4314/iijikm.v12i1.3

[ref60] Wright K. B. (2005). Researching internet-based populations: advantages and disadvantages of online survey research, online questionnaire authoring software packages, and web survey services. J. Comput.-Mediat. Commun. 10:JCMC1034. doi: 10.1111/j.1083-6101.2005.tb00259.x

[ref61] Wu W. Li Y. Li H. Zhang T. (2022). Perceived environmental corporate social responsibility and employees' innovative behavior: a stimulus-organism-response perspective. Front. Psychol. 12:777657. doi: 10.3389/fpsyg.2021.777657, PMID: 35173648 PMC8841478

[ref62] Xu C. Tu C. C. (2022). Impact of college students’ learning adaptation on learning conformity behavior in Hengyang: moderating role of peer attachment. Asia-Pacific Edu. Res. doi: 10.1007/s40299-022-00678-x

[ref03] Yongmei L. Xuhua W. Xiaohong C. (2011). The relationship among emotional intelligence, conflict management, and perceived cohesion. Science Research Management. 32, 88–96.

[ref64] Zhang Y. He B. Sun X. (2018). The contagion of unethical pro-organizational behavior: From leaders to followers. Front. Psychol. 9:1102. doi: 10.3389/fpsyg.2018.01102, PMID: 30018583 PMC6038011

